# Genomic diversity study of highly crossbred cattle population in a Low and Middle Tropical environment

**DOI:** 10.1007/s11250-024-04011-0

**Published:** 2024-09-18

**Authors:** Luisa Fernanda Naranjo Guerrero, Andrés Rogberg-Muñoz, Nancy Rodríguez, Luis Gabriel González Herrera

**Affiliations:** 1 Grupo de investigación GIPAB, sede Medellín, Colombia; 2 Universidad Nacional de Colombia, sede Medellín, Colombia; 3 Universidad de Buenos Aires, Facultad de Agronomía. Cátedra de Mejoramiento Genético Animal, Buenos Aires, Argentina; 4 CONICET – Universidad de Buenos Aires, Instituto de Investigaciones en Producción Animal (INPA), Buenos Aires, Argentina; 5 Universidad Francisco de Paula Santander, seccional Ocaña, Colombia; 6 Grupo de Investigación en Biodiversidad y Genética Molecular (BIOGEM), Universidad Nacional de Colombia, sede Medellín, Colombia

**Keywords:** Genetic structure, Genomic, Linkage Disequilibrium, Milk production

## Abstract

Milk production in tropical regions plays a crucial role both economically and socially. Typically, animals are utilized for dual purposes and are genetically obtained by an intense crossbreeding between Zebu and/or locally adapted breeds, alongside specialized breeds for dairy production. However, uncontrolled mating and crossbreeding may affect the establishment of an effective animal breeding program. The objective of this study was to evaluate Genomic diversity of highly crossbred cattle population in a Low and Middle Tropical environment. All sampled animals were genotyped using the Genessek GGP Bovine 100 chip (n = 859) and public genomic information from eight breeds were employed as reference. The genetic structure of the population was estimated using a Principal Component, Bayesian clustering and a linkage disequilibrium analysis. PCA results revealed that PC1 explained 44.39% of the variation, associated with the *indicus/taurus* differentiation, and PC2 explained 14.6% of the variation, attributed to the differentiation of Creole and European components. This analysis underscored a low population structure, attributed to the absence of genealogical tracking and the implementation of non-directed crossbreeding. The clustering shows an average contribution of Zebu, Creole, and European Taurine components in the population was 53.26%, 27.60%, and 19.13%, respectively. While an average LD of 0.096 was obtained for a maximum distance of 400 kb. The LD value was low in this population, probably due to the almost no selection applied and the recombination events that occurred during its development. These findings underscore the value of crossbreeding in tropical dairy production but emphasize the importance of directing the mattings.

## Introduction

Milk production in the tropics plays a pivotal role for economic and social development. This activity contributes to income diversification for producers, efficient utilization of resources, food nutritional security for the population and the generation of employment in rural areas. Furthermore, tropical dairy production usually combines a dual-purpose system, either as simultaneous milk and meat production or using animals for transportation or work. These are common strategies of medium and small producers to enhance profitability and resilience in the face of market changes (Brito et al. 2021).

In Colombia and other tropical countries, most animals used in dual-purpose systems result from various crosses between Zebu breeds (*Bos primigenius indicus*) and specialized milk breeds such as Holstein, Normande, Brown Swiss (*Bos primigenius taurus*), and in some regions, with native taurine breeds (Dane 2015). Most producers utilize crossbreeding looking for a breed composition that is best adapted to the specific climatic conditions of each region, thereby enabling the attainment of desired productive and reproductive parameters. Nowadays, due to the challenges posed by climate change, various strategies have been proposed to mitigate its impact, some of which focus directly on animal breeding by strengthening local breeds and crossing animals adapted to specific environmental conditions (Pinto Díaz and Rojas Peña 2022). However, the breed composition of most animals used is partially or completely unknown (Elzo 2011). This situation requires significant attention because the use of a wide variety of breeds and crosses, the lack of records (productive, reproductive, and health), and the use of non-directed mating could result in less productive individuals that are poorly adapted to the specific environmental conditions of each region.

With the advent of genotyping techniques, reliable analyses have been developed to estimate the genetic structure and relationships between populations in both, purebred and crossbred cattle (Mastrangelo et al. 2018). These techniques have enabled the examination of individuals’ genome to determine their genetic and breed composition (Rosero Alpala et al. 2021). Furthermore, these techniques have also allowed the identification of genetic markers associated with economically important traits, facilitating the selection of animals with desirable traits in animal breeding programs.

The objective of this study was to evaluate Genomic diversity of highly crossbred cattle population in a Low and Middle Tropical environment, to generate information on the genetic structure for a breeding program that enables the performance of productive animals adapted to the tropics with greater resilience to climate change.

## Materials and methods

### Study population and sampling

The study population consisted of a total of 859 crossbred animals from different herds (up to 10 animals per herd) dedicated to milk production in low and middle tropical conditions across various regions of Colombia (Antioquia, Boyacá, Caldas, Caquetá, Cesar, Cundinamarca, Magdalena, Quindío, Risaralda, Santander, Tolima, and Valle del Cauca). These areas exhibit diverse agroecological and climatic conditions, ranging in altitude from 130 to 2000 meters above sea level, with average temperatures ranging from 18°C to 30°C and annual precipitation ranging between 500 and 3000 mm; however, the animals originate from locations with a maximum altitude of 1500 meters above sea level. A tail hair sample (containing 10–20 hairs) was collected, ensuring the cleanliness of the extraction area to eliminate potential contaminants. Samples were stored on FTA Classic® WHATMAN cards and paper envelopes, and later kept at room temperature until delivery to the NEOGEN® laboratory (http://www.neogen.com), responsible for DNA extraction and sample genotyping.

### Genotyping and quality control

All sampled animals were genotyped using the Genessek GGP Bovine 100 chip (~100,000 SNPs). Genomic information was analyzed using Plink V1.9 (Purcell et al. 2007). Initially, insertions and deletions (INDELS), mitochondrial SNPs, those associated with sex chromosomes, with unknown positions, or duplicated within a chromosome were removed, resulting in a total of 89,761 SNPs for the subsequent stage. Quality control for SNPs included a call rate > 90% and adherence to Hardy-Weinberg equilibrium (HWE) > 0.00001. After quality control, the total number of SNPs analyzed for the population was 86,499 with 855 animals. The mean call rate per SNP was 0.9964, and per individual was 0.9840. The minor allele frequency (MAF) was 0.2779.

### Reference population

To encompass breeds that might have contributed to the genetic background of the sampled animals or were related to them, the public WIDDE cattle database (ide.toulouse.inra.fr/ide) was utilized. Genomic information from 8 reference populations genotyped with the Illumina BovineSNP50v1 and Illumina BovineSNP50v2 chips was obtained. Reference breeds were selected based on historical data and phenotypic assessment in the different farms, including European highly selected dairy breeds as Holstein (HOL), Jersey (JER) and Brown Swiss (BSW), and the Zebu breeds Gyr and Brahman (BRH). As only genotypes from Colombian Creole breed Romosinuano were available, the data from Guadalupe Creole and Senepol were also included, to identify possible common ancestral Creole breed components present in the sampled animals. Both breeds are creole breeds from Guadalupe in the French Antilles and the Caribbean Island of Saint Croix, respectively, which are phylogenetically close to Colombian Creole and have demonstrated adaptation processes to tropical environments (Naves et al. 2005; Huson et al. 2014; Raschia and Poli 2021). For the analysis, only the SNPs that were common between the study population (TEST) and the reference population were used.

### Population and individual genetic structure

The origin and genetic relationships of different individuals in the population were determined through two complementary analyses of population structure. Initially, the genetic diversity in the population was assessed using Principal Component Analysis (PCA) with PLINK V1.90 (Purcell et al. 2007) and R v4.0.3 (R Core Team 2021). This analysis determined genetic distances between individuals and potential dispersions among the genetic groups established in the study population and the pure breeds used as reference. Subsequently, the software Structure V2.3.4 (Sthephens and Donelly 2000) was employed to estimate the individual breed composition within the study population and its relation to the reference breeds. The most probable number of populations (k) was estimated by running 5 executions for each k (k = 3 – 9) with a burn-in length of 10,000 and 100,000 MCMC. The optimal k value was selected using the method proposed by Evanno et al. (2005), based on the difference in likelihood between runs (∆K). A clear peak in the true value of k, i.e., the k with the highest ∆K value, was chosen as the optimal k. Additionally, other values of k were evaluated taking into account the historical information and the reference populations used.

### Linkage Disequilibrium (LD) analysis

Linkage Disequilibrium (LD) can be employed to assess genomic mixing processes, such as crossbreeding. In recent mixing processes, larger conserved segments are observed, while successive recombination across several generations result in the reduction of the length of those segments. In this context, LD values were estimated using the r^2^ statistic between all pairs of SNPs within each chromosome for the population. LD estimations covered distances from 0 to 400 kb using the PLINK v1.90 program. The LD values were grouped into intervals: <25 kb, 25-50 kb, 50-100 kb, 100-200 kb, 200-400 kb with the aim of capturing recent admixture in the population. Subsequently, these values were graphed to observe the decay of linkage disequilibrium. The calculation of LD using the r^2^ statistic was proposed by Hill and Robertson (1968).

$${r}^{2}=\frac{freq\left(A1B1\right)*freq\left(A1B2\right)*freq\left(A2B1\right)*freq\left(A2B2\right)}{freq\left(A1\right)*freq\left(A2\right)*freq\left(B1\right)*freq\left(B2\right)}$$

In this context, the SNP “A” is considered with alleles A1 and A2, and SNP “B” with alleles B1 and B2, along with haplotypic frequencies freq(A1B1), freq(A1B2), freq(A2B1), and freq(A2B2)

## Results

### Population diversity through principal component analysis

**Fig. 1:**
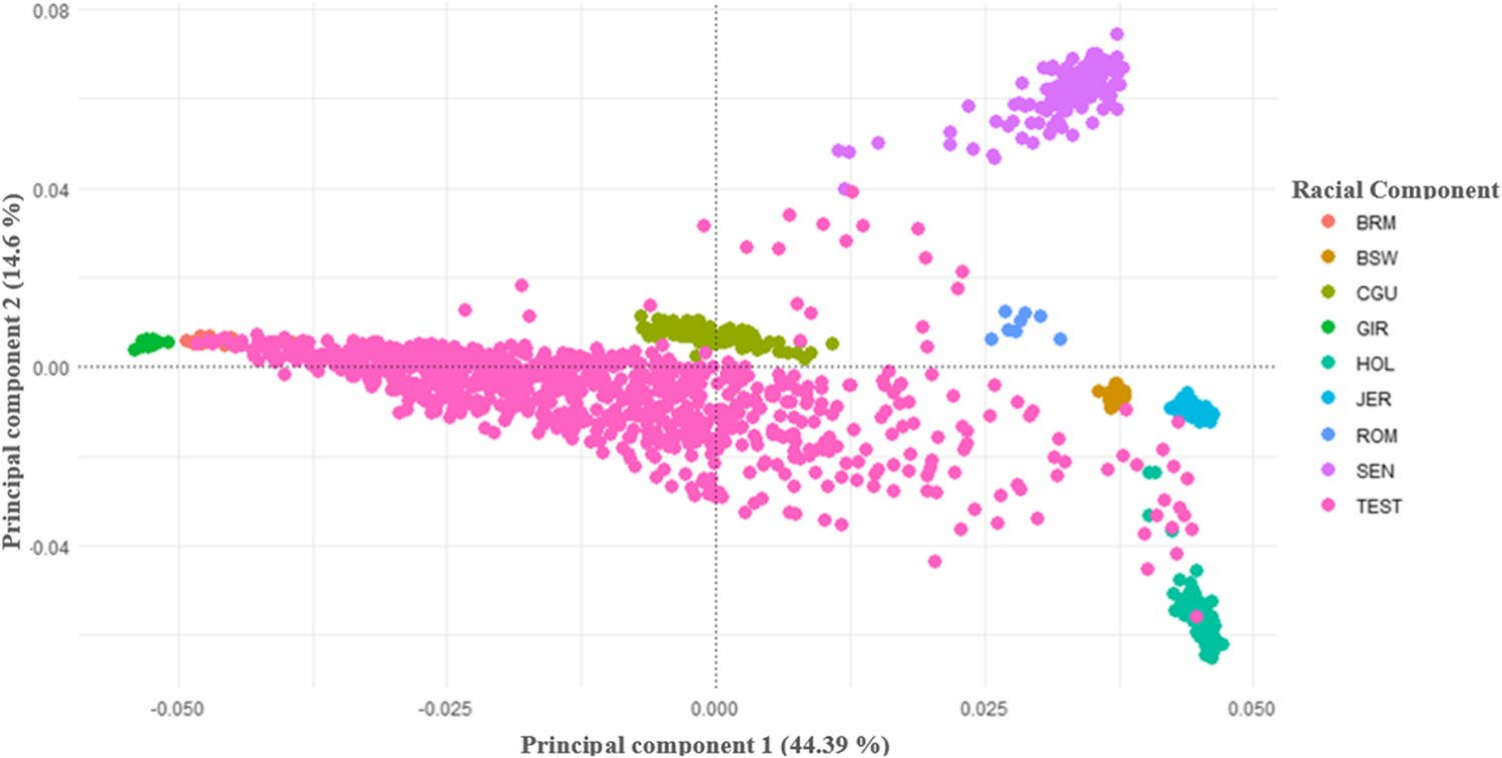
Principal Component Analysis of the study Population (Test) Including Reference Groups. BRM: Brahman, BSW: Brown Swiss, CGU: Criollo Guadalupe, GIR: Gyr, HOL: Holstein, JER: Jersey, ROM: Romosinuano, SEN: Senepol, TEST: Crossbred study population

Genomic information was leveraged to assess the genetic diversity within the sampled crossbreed population, employing Principal Component Analysis (PCA). This analysis incorporated individuals from pure reference breeds, as illustrated in Fig. 1. The highly selected breeds (BRM, BSW, GIR, HOL, JER) exhibit lower dispersion in the graph which suggests a greater genetic homogeneity. In contrast, the Creole breeds (CGU, SEN, ROM) and the studied population (TEST) display higher dispersion, explaining a greater genetic variability. However, the dispersion observed in the Creole breeds is overshadowed by the significant dispersion observed in the studied population (TEST). Principal Component 1 explained 44.39% of the variation, associated with genomic differentiation between animals of the *Bos primigenius indicus* group (BRM and GIR) positioned on the left side of Fig. 1, and *Bos primigenius taurus* group (BSW, HOL, JER, ROM, SEN) located on the right side of Fig. 1. The hybrid origin (zebuine and taurine) of the CGU breed in the TEST population can be evidenced in the center of the graph, with a wide dispersion of observations.

The second principal component explained 14.6% of the variation and reveals the variation within the *Bos primigenius taurus* group. The animals that showed a higher presence of Creole component (CGU, ROM, SEN) were positioned at the top of Fig. 1, while animals with a higher presence of European component (HOL, JER, BSW) were located at the bottom of Fig. 1. Additionally, the also great dispersion of the studied population in this axis reveals the multiplicity of lineages used for crossbreeding.

#### Analysis of individual genetic composition and genetic relationship between populations

**Fig. 2:**
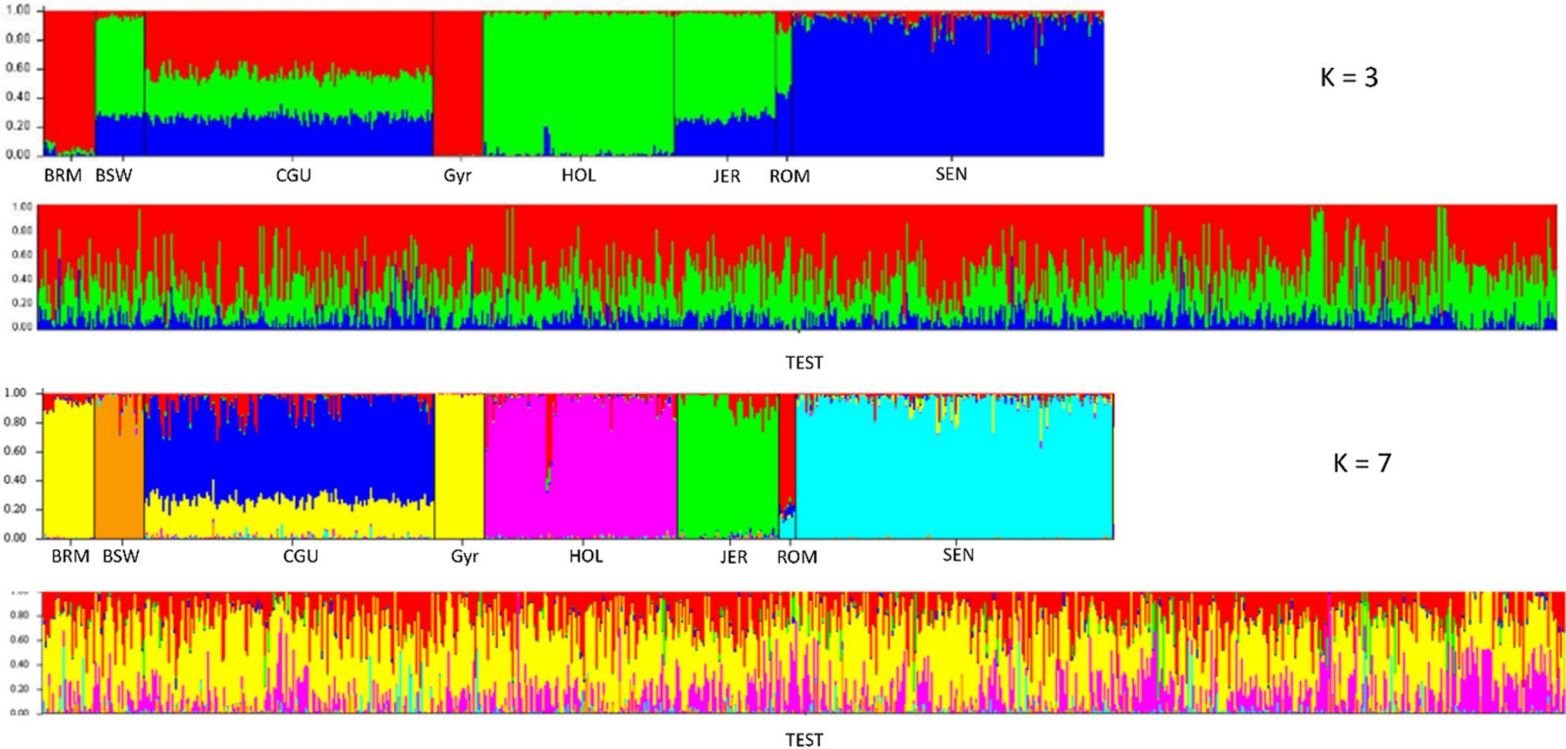
Population Structure results in the sampled population and reference breeds. In the graph, each individual is represented by a vertical line, and the color corresponds to a different genetic component. For k = 3, Zebu: red, European: green, and Iberian: blue. For k = 7, Zebu: yellow, Brown Swiss: orange, Creole Guadalupe: blue, Holstein: pink, Jersey: green, Romosinuano: red, Senepol: light blue

While PCA analysis allows for low-dimensional projections of data that explain considerable variation in genotypes, it does not directly provide mixing fractions. This was addressed by the clustering analysis performed with Structure program. The results for k = 3 and k = 7 are shown in Fig. 2. Using the method of Evanno et al. (2005), k = 3 was determined as the ancestral genetic structure with the lowest validation error separating Zebu (red) and two taurine (green and blue) components. Examining the breeds with a taurine ancestral lineage, there is a “continental European” component (green), primarily detected in Holstein but shared with Brown Swiss, Jersey, Romosinuano, and Criollo Guadalupe. Another component that can be considered is an “Iberian taurine” (blue), mainly detected in Senepol and shared with Brown Swiss, Jersey, Romosinuano, and Creole Guadalupe (Fig. 2). At k = 3, the Colombian population appears as a mixture of the three lineages, consistent with historical data indicating a Creole base with successive crossbreeding with European and Zebu dairy breeds.

Then, the rest of the results for other k values were analyzed; from this, and considering the historical data, it was observed that the result for k = 7 correctly differentiated the breeds included as reference (Fig. 2). It can be observed that the crossbreed animals predominantly exhibit a mixed genetic composition with a Zebu component (yellow), a Holstein component (pink), and a third Creole component (red) shared with Romosinuano. Additionally, the results show that some animals presented Brown Swiss (orange) and Jersey (green) ancestry. On the other hand, in this k, three Creole components can be observed: i) the aforementioned one (red) shared with Romosinuano, which is also present in low proportion in Guadalupe Creole and Senepol, ii) Guadalupe Creole component (blue), partly shared with Romosinuano and present in very low frequency in the Colombian crossbreed population; and iii) Senepol component (light blue), also shared with Romosinuano and observed in some individuals in the study population.

Based on the information obtained through the structure analysis at k = 7, it was decided to form three genetic groups by summing the different ancestral components. The grouping was done as follows, establishing three genetic groups: i) Zebu, ii) Creole (sum of components CGU, ROM, and SEN), and iii) European Taurine (sum of components HOL, JER, BSW). Under this grouping, it was found that the Zebu and Creole components represents the highest proportion in the mixed population of these Colombian areas. The average Zebu contribution was 53.26%, ranging from 1% to 93.40%; the average Creole contribution was 27.60%, ranging from 5.00% to 85.20%; whereas and the average European Taurine contribution was 19.13%, ranging from 3.00% to 96.00%.

It was observed that for the Creole component, there is a higher genetic contribution from the component shared with ROM, which is coincident with a phylogeographical perspective. For the European Taurine component, the greater contribution was represented by the HOL breed, which coincides with historical and phenotypic data observed in the sampled animals (Table 1). On the other hand, the study population showed varied breed proportions, demonstrating again the extent of admixture and generated diversity.

**Table 1:** Average breed contribution in percentage in crossbreed sampled study population concerning the genetic group of control breeds (k = 7)

Component	Reference Breed Group	Genetic contribution		
		Average	Minimum	Maximum
Zebu	Zebu	53.26 %	1.00%	93.40%
Creole	CGU	1.18 %	5.00%	85.20%
	ROM	24.59 %		
	SEN	1.82 %		
European Taurine	HOL	14.77 %	3.00%	96.00%
	JER	2.21 %		
	BSW	2.14 %		

*Zebu* Brahman and Gyr, *CGU* Guadalupe Creole, *ROM* Romosinuano, *SEN* Senepol, *HOL* Holstein, *JER* Jersey, *BSW* Brown Swiss

#### Linkage Disequilibrium (LD) analysis (r^2^)

For this study, a mean linkage disequilibrium (r^2^) of 0.096 was obtained for a maximum distance of 400 kb. Similarly, the lowest r^2^ was observed on autosome 25 (BTA 25) with a value of 0.078, and the highest r^2^ value was observed on autosome 5 (BTA 5) with values of 0.107 (Table 2).

**Table 2:** Estimated r^2^ values for each autosome (BTA) in the population

BTA	SNP (n)	r^2^	SD
1	5286	0.102	0.160
2	4484	0.099	0.154
3	4292	0.101	0.160
4	3839	0.093	0.146
5	4283	0.107	0.168
6	4133	0.093	0.156
7	3682	0.100	0.163
8	3636	0.102	0.157
9	3544	0.098	0.157
10	3473	0.094	0.150
11	3618	0.097	0.153
12	2885	0.092	0.146
13	2911	0.098	0.153
14	2888	0.103	0.160
15	2915	0.093	0.152
16	2684	0.100	0.161
17	2541	0.086	0.142
18	2447	0.090	0.152
19	2505	0.101	0.162
20	2622	0.099	0.165
21	2447	0.100	0.160
22	2111	0.087	0.143
23	1977	0.093	0.157
24	2150	0.097	0.150
25	1621	0.078	0.137
26	1735	0.082	0.141
27	1624	0.080	0.138
28	1629	0.078	0.133
29	1761	0.088	0.140

*BTA* Autosomes; *r*^2^ Linkage disequilibrium estimate, *SD* standard deviation

Additionally, Table 3 shows the average linkage disequilibrium values for each marker pair at distances up to 400 kb and the percentage of marker pairs with an *r*^2^ value greater than 0.30%.

**Table 3:** Average linkage disequilibrium for marker pairs separated by up to 400 kb, according to *r*^2^ statistics and the percentage of marker pairs with *r*^2^ > 0.30

Distance (kb)	r^2^	SD	Number of SNPs with r^2^ > 0.30	Percentage of SNPs with r^2^ > 0.30
25	0.240	0.272	20363	28.17
50	0.146	0.195	12138	15.37
100	0.100	0.147	12475	8.44
200	0.068	0.104	9954	3.79
400	0.055	0.083	3818	1.99

*SD* standard deviation; *r*^2^ Linkage Disequilibrium Estimation

**Fig. 3:**
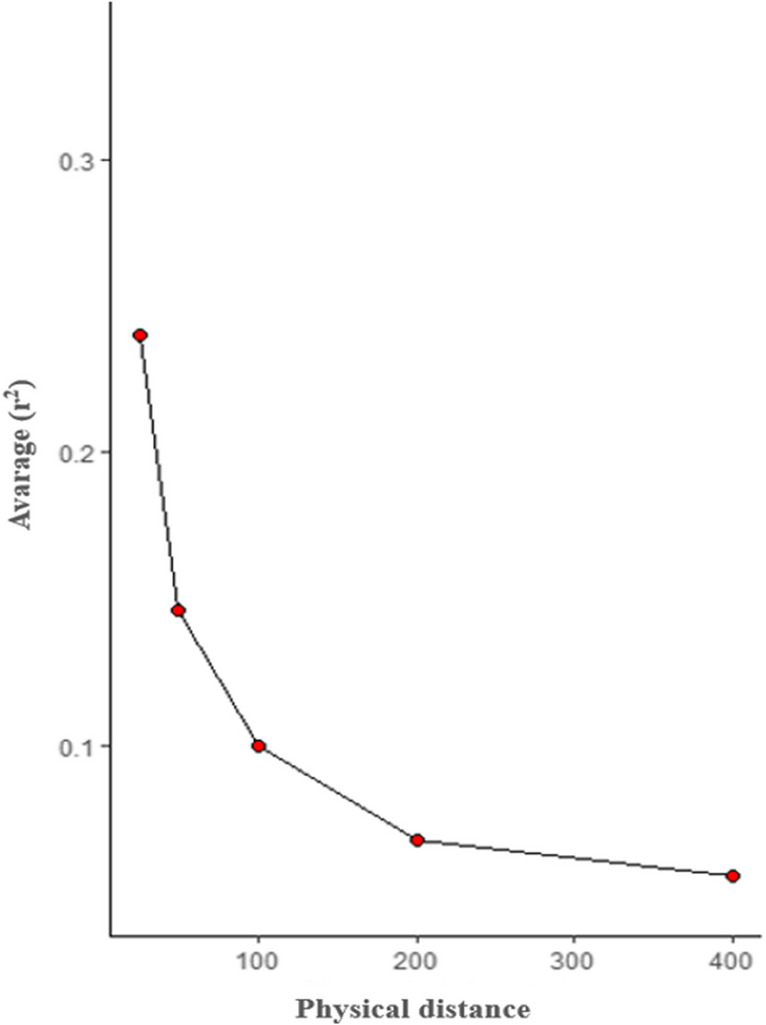
Displays the average *r*^2^ values for different distance groups, showing a decrease in the *r*^2^ values as the distance between marker pairs increases, indicating the decay of linkage disequilibrium

Finally, in Fig. 3 displays the decay of linkage disequilibrium.

## Discussion

### Population diversity through principal component analysis

The Principal Component Analysis conducted using pure breeds as references revealed a limited population structure, confirming the presence of multiple events of crossbreeding in the population (TEST). These results are, in part, a consequence of indiscriminate crossbreeding and the lack of genealogical tracking, leading to individuals with undetermined or undefined breed backgrounds. Such crossbreeding practices generate genetic instability, hindering the phenotypic and productive expression of a particular population (Martinez Tovar et al. 2017). This instability also interferes with the implementation of animal breeding programs. In this regard, a recent study conducted on dual-purpose herds in Colombia observed a similar pattern in the evaluated crossbred animal population (Rosero Alpala et al. 2021).

When controlled crossbreeding occurs between breeds, it is expected that the allele frequencies of the animals will be related to the degree of crossbreeding between original pure breeds and the generation from the admixing event (Bolormaa et al. 2011). Taurine and Zebu crossbreeding has been widely done to exploit the hybrid vigor (non-additive effects); whereas the further selection could take advantage of complimentary (additive effects) (Goszczynski et al. 2018). However, in dual-purpose production systems, crossbreeding tends to be conducted without considering breed proportions, in the pursuit of obtaining animals that are more adapted and exhibit higher reproductive and productive efficiency. Nevertheless, this hybrid vigor could be lost without proper tracking of matings, resulting in less productive individuals with lower reproductive rates (Zambrano et al. 2013).

## Analysis of individual genetic composition and genetic relationship between populations

In tropical dairy production a breed proportion greater than 50% of *Bos primigenius taurus* is associated with a higher milk volume and a reduced reproductive response, whereas a higher proportion of *Bos primigenius indicus* is linked to greater adaptability and superior reproductive indices (Zambrano et al. 2013; Perdomo et al. 2017). This has encouraged crossbreeding between Zebu and taurine animals trying to obtain productivity, good reproduction, and adaptation. However, the execution of unguided crossbreeding could result in animals with low productive and reproductive indices, which have been seen many times in this kind of schemes (Perdomo et al. 2017). Even though, the actual research has shown that seems to be a need of a minimum proportion of adapted genome (Zebu or Creole) to assure an adaptation. The population structure analysis identified an optimal *k* = 3, corresponding to the Zebuine, European taurine, and Creole (Iberian taurine) lineages, and the sum of the average contributions indicated that animals, on average, have a 80.85% breed contribution adapted to the tropical environment (53.26% Zebu + 27.59% Creole). Effectively, the *k* = 7 has demonstrated the relationships with the reference breeds, providing insights into the practice of multiracial crossbreeding among breeders.

This study successfully demonstrated a pronounced genetic diversity within the studied population, revealing significant genetic variations. These findings extend beyond this specific group, as evidenced by research on animals used in dual-purpose productions (Rosero Alpala et al. 2021). The authors argue that the high genetic diversity in these populations can be attributed to the lack of genealogical tracking and the implementation of non-directed crosses. Moreover, they contend that the extensive array of crossbreeding and the prevalence of the indicus phenotype are grounded in the pursuit of complementing attributes associated with high milk production, typically found in taurus-type dairy breeds. However, despite high the Zebu and Creole components proportions observed, only a quarter of the animals exhibit a taurine European component higher than 30% which highlight the need for a significant contribution from an "adapted genome" for productive viability.

## Linkage Disequilibrium (LD) analysis (r^2^)

The extent of the LD present in a population serves as an indicator for historical recombination events, allowing inferences about genetic diversity, geographical subdivision, and genomic regions that have undergone selection (McKay et al. 2007; Slatkin 2016). The estimation of LD values varies depending on the population, marker type, sample size, and chip density used in each study. For example, Salem et al. (2018) obtained higher LD values for BTA 14 in Holstein cattle, while Lu et al. (2012) found higher LD values in BTA 5 in Angus beef cattle. These autosomes also showed higher LD in the crossbred population of this study with values of 0.1066 (BTA 5) and 0.1032 (BTA 14). Several QTL and genes affecting traits such as birth weight and carcass traits, including IGF-1 and myf5, have been identified on BTA 5. On BTA 14, genes affecting milk yield, such as DGAT1, have been observed (Akçay et al. 2020). Therefore, selection to improve birth weight, carcass traits, and milk production could increase LD on BTA 5 and BTA 14. On the other hand, in Nellore cattle, the lowest LD values were found for BTA 1, BTA 27, BTA 28, and BTA 29 (Espigolan et al. 2013), which aligns with the results of this study where BTA 27 and 28 were the autosomes with the lowest LD values.

Only markers separated by up to 25 kb showed an average r^2^ above 0.20 – 0.30 (Table 3), a range typically used in previous studies to define LD levels for genomic selection methodologies (Hayes et al. 2009). Neves et al. (2015) had found average r^2^ values in this range at distances up to 100 kb in Gyr dairy cattle, Martínez-Reina et al. (2020) up to 33 kb in Simmental cattle, Salem et al. (2018) up to 70 kb in Holstein cattle, Bejarano et al. (2018) up to 200 kb in BON and Romosinuano cattle, and Lu et al. (2012) up to 70 kb in Angus and 30 kb in Charolais and crossbreds. The short distance observed demonstrated the lack of uniformity in the population probably due to the extensive crossbreeding, which suggests an extra difficulty when thinking about implementing an animal breeding program through selection for this kind of populations, as the models to be used should consider the multibreed genomic complexity.

It has been described that LD is influenced by factors such as genetic drift, crossbreeding, mutation and recombination rates, selection, population size, bottleneck events, and other genetic events that a population may undergo (Qanbari 2020). In the case of crossbreeding, it has been noted that mating between populations creates significant LD values, which depend on the similarity in allelic frequency profiles present in the populations being crossed. Thus, crossing between endogamous populations generates significant LD. However, this value could be small if populations with similar genetic frequencies were crossed, and these frequencies deteriorate rapidly, disappearing after a limited number of generations (Qanbari 2020). Regarding the findings in the crossbred population, Lu et al. (2012) reported a similar behavior to that found in this study for crossbred animals, where LD values were lower than those observed in pure Angus and Charolais breeds. These findings align with the diverse origins and extensive crossbreeding practices observed in these cattle populations, posing a challenge for the implementation of improvement programs in such heterogeneous genetic pools. Lastly, the low LD values obtained may be attributed to a minimal or absent selection and several potential recombination events involving multiple breeds in their genetic background.

## Conclusion

In conclusion, our study delves into the genetic ancestry and genome structuration of a highly admixed population utilized for dairy production in the Low and Middle tropics of Colombia. The results demonstrate admixing between Zebu, Creole, and European taurine genomes. However, only a quarter of the animals exhibit a value higher than 30% of the eldest, emphasizing the necessity of a significant contribution from an "adapted genome" for productive viability in the tropics. Finally, the LD analysis reveals a reduced LD extension, implying challenges in establishing an animal breeding program. Taken together, these findings underscore the value of crossbreeding in tropical dairy production but emphasize the importance of controlling the matings.

## Data Availability

The data for this study are available from the corresponding author upon request.
